# From IMPACT to RECLAIM: A Single-Stage Cell Therapy for Articular Cartilage Repair and a Platform for Musculoskeletal Tissue Regeneration

**DOI:** 10.1007/s12178-025-09949-8

**Published:** 2025-02-11

**Authors:** Christopher V. Nagelli, Jasmijn V. Korpershoek, Katy Lydon, Lucienne Vonk, Roel Custers, Aaron J. Krych, Daniel Saris

**Affiliations:** 1 Department of Orthopedic Surgery, Division of Sports Medicine, Mayo Clinic, 200 First Street SW, Rochester, MN 55905 USA; 2 Department of Orthopaedics, University Medical Center Utrecht, Utrecht, The Netherlands

## Abstract

**Purpose of Review:**

Cartilage injury does not heal spontaneously. The current cell-based cartilage treatments have either demonstrated poor clinical outcomes, require two surgeries, or are costly and logistically challenging. To overcome these challenges, our team has developed a one-stage, two cell-type surgical cell therapy for acute chondral defects. This procedure combines allogeneic mesenchymal stromal cells (MSCs) and autologous chondrons to harness MSCs as signaling cells to stimulate chondrons to promote tissue repair. This procedure has been investigated in clinical trials conducted in both Europe and the United States which are called IMPACT and RECLAIM, respectively. This article provides a review of our preclinical and clinical research which led to the development of this cell therapy.

**Recent Findings:**

The combination of allogeneic MSCs and autologous chondrons in preclinical studies have demonstrated to synergistically stimulate cartilage repair, and the combination of cells outperforms either cell-type alone. In clinical trials, the combined cell therapy was safe to use, improved knee function, and demonstrated durable pain reduction.

**Summary:**

Our single-stage, combined cell therapy of allogeneic MSCs and autologous chondrons is a viable cell therapy for acute articular cartilage defects. We anticipate this combined cell therapy may be a platform cell therapy for a wide range of musculoskeletal repair applications.

## Introduction

Acute injuries to articular cartilage within synovial joints are common among young, active patients. Cartilage injuries can be painful, cause dysfunction, and decrease quality of life. Because cartilage has no intrinsic repair capacity, cartilage lesions require surgical intervention [1]. The two most common cell-based procedures are microfracture and autologous chondrocyte implantation (ACI) [2]. Microfracture is typically indicated for smaller defects up to 2 cm^2^. The microfracture surgical technique initiates channels between the osseous layer and the underlying bone marrow to allow the marrow, containing mesenchymal stromal cells (MSCs), to fill the defect area, form a fibrin clot, and initiate tissue repair. However, the repair tissue resulting from microfracture resembles fibrocartilaginous scar tissue rather than native articular cartilage, as demonstrated in long-term clinical studies [3, 4]. Larger defects are commonly treated with autologous chondrocyte implantation (ACI), a two-step surgical procedure [5]. During the first surgery, cartilage is harvested using a small biopsy, from which chondrocytes are isolated and culture expanded in a Good Manufacturing Practice facility. The expanded chondrocytes are implanted back into the defect in a second surgical procedure 5–12 weeks later. If the cells are loaded onto a scaffold material, the procedure is called matrix-assisted ACI (MACI). These procedures have led to improvements in patient reported outcomes and low rates of revision or reoperation at 10 years follow up [6]. However, the two-stage surgical procedure significantly increases patient burden. In addition, the cell expansion delays rehabilitation, results in exceedingly high costs, and can lead to dedifferentiation of chondrocytes. To overcome these challenges, our team has developed a one-stage cell-based surgical procedure for acute cartilage defects [7]. 

**Fig. 1:**
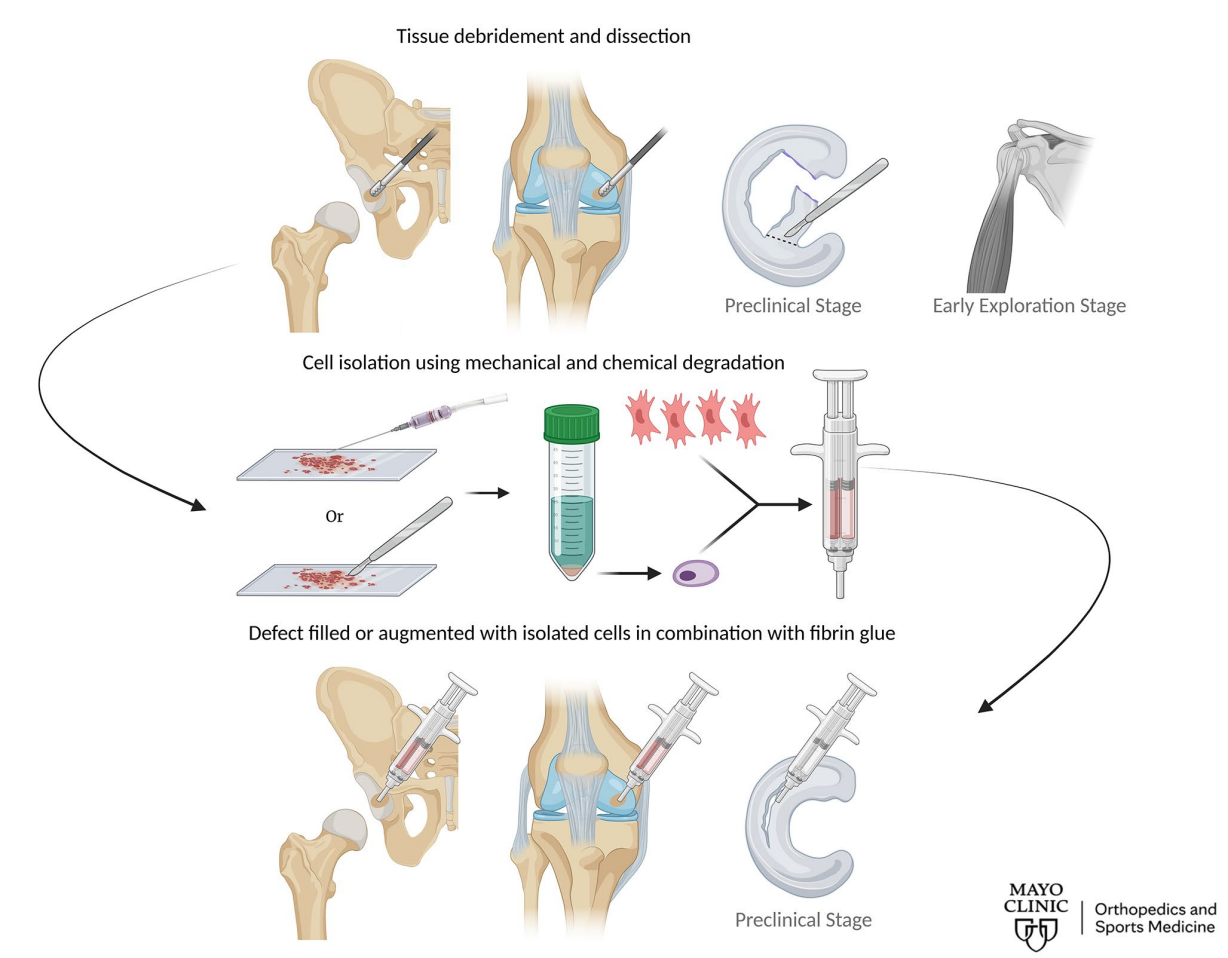
Hip and knee RECLAIM are currently used in clinic, while RECLAIM for meniscus is in a preclinical stage of research. The application of RECLAIM to rotator cuff injuries is currently being explored

The fundamental principle of our cell therapy is to combine two cell-types, autologous chondrons and allogeneic MSCs, to harness MSCs as signaling cells to stimulate endogenous tissue repair by chondrons. The use of allogeneic MSCs is critical because it allows us to implement a one-stage, cell therapy surgical procedure since only a limited number of chondrons can be isolated. This indicates that our cell therapy can be implemented at reduced costs because the MSCs can be pre-expanded this allows them to be available as an off-the-shelf product [8]. We have conducted a decade long line of basic science and clinical research which includes in vitro cell cultures, preclinical animal models, and clinical trials to develop this combined treatment [7, 9, 10]. Most of this work began with our research team at the University Medical Center Utrecht located in The Netherlands, who were first to combine bone-marrow derived MSCs and autologous chondrons. We termed this work IMPACT because it was an *I*nstant *M*SC *P*roduct accompanying *A*utologous *C*hondron *T*ransplantation [11]. Subsequently, we developed an allogeneic MSC bank at Mayo Clinic in Rochester and extended both our basic science research and clinical trial using adipose-derived MSCs, instead of bone marrow-derived MSCs. In addition, to verify adipose-derived MSCs behaved in a similar way as stimulatory signaling cells resulting in approximately equal outcomes, we repeated the human clinical trial at Mayo Clinic with adipose-derived MSCs and autologous chondrons. This was called RECLAIM because we use *RE*cycled *C*arti*L*age from the defect area, combine *A*utologous chondrons and Allogeneic MSCs, and *Im*plant them back into the defect. Much like ACI and MACI, IMPACT and RECLAIM cell therapies are considered Advanced Therapy Medicinal Products. A schematic of the IMPACT and RECLAIM surgical procedure is shown in Fig. 1.

In this review, our goal is to summarize our preclinical and clinical research on IMPACT and RECLAIM cell therapy, suggest a possible mechanism underpinning our outcomes, and discuss how this combined cell therapy can be used as a platform for other injured or diseased musculoskeletal tissues. The research discussed here uses bone-marrow derived MSCs, unless otherwise stated.

### Current Evidence on Combined Cell Therapy

#### Preclinical Studies

Early preclinical studies focused on identifying the specific cell-type or combination of cells which can stimulate articular cartilage repair within the cartilage defect. The ACI procedure relies on chondrocytes to facilitate this repair, however, because of the low cellularity of the harvested cartilage biopsy, culture expansion of these cells is required. As mentioned previously, there are several disadvantages to this approach including an additional surgical step which raises costs, increases the possibility of infection, dedifferentiation of the chondrocytes, and further delays a return to activity. One possible solution would be to include another cell type to stimulate the isolated chondrocytes and eliminate the additional culture expansion step. Earlier studies have demonstrated that combining chondrocytes with MSCs can stimulate the production of cartilage matrix and the upregulation of chondrogenic genes [12–14]. Chondrocytes reside within articular cartilage surrounded by a pericellular matrix. The chondrocyte together with the pericellular matrix is referred to as a chondron and makes up the structural and functional unit of the tissue. The pericellular matrix consists mainly of structural proteins such as collagen type VI, decorin, and fibronectin which are important for the cell’s metabolic activity and mechano-signaling. The (M)ACI procedures remove this matrix during the cell isolation and expansion, thereby altering the fundamental behavior of the cell. Moreover, chondrons perform better than chondrocytes in terms of articular cartilage matrix molecule production and the pericellular matrix protects against collagen-mediated upregulation of MMPs [15–18]. Therefore, we evaluated the chondrogenic potential of combining chondrons and MSCs during preclinical studies [7]. Using a pellet co-culture model, cartilage formation was compared between chondrons and MSCs or chondrocytes and MSCs [7]. MSCs stimulated cartilage specific matrix production in a dose-dependent fashion, and the effect was greater in co-cultures with chondrons than with chondrocytes [7]. These findings indicated that combining chondrons and MSCs may promote the endogenous repair of articular cartilage.

To establish feasibility of a one-stage approach, chondrons must be isolated consistently and rapidly from the articular cartilage matrix [19]. We demonstrated this with a rapid chondron digestion protocol that was developed in our lab [19]. With this protocol, we can isolate 1.3 million chondons per gram of recycled cartilage from within the patient’s defect area within 40 min [19]. This protocol has been validated and reproducibility has been demonstrated with articular cartilage from several species, including human, equine, porcine, and bovine [19]. Furthermore, an important aspect of this is that we worked with Good Manufacturing Practice (GMP)-suitable raw materials, substances, and disposables which allowed for more rapid translational. Along this journey, we developed processes, wrote standard operating procedures, and established criteria for its successful clinical implementation [7, 11, 19]. These steps were critical for transitioning this technique into the clinic as a one-stage procedure.

### Preclinical Animal Models

The optimal ratio of MSCs and chondrons was established in vivo using preclinical animal studies. A total of 250,000 cells were subcutaneously implanted in nude mice in various ratios of chondrons to MSCs enclosed within a fibrin scaffold (0:100, 5:95, 10:90, 20:80, chondrons to MSCs) [7]. As a control group, a fibrin scaffold of chondrons only was used [7]. After 4 weeks, a significantly higher GAG content was found in the 20:80 to 10:90 chondron/MSC scaffold compared to the chondron-only scaffolds, indicating that the MSCs stimulated production of cartilage-specific matrix when cultured together with chondrons [7]. Even after correcting for the amount of DNA present after 4 weeks, the 10–20% chondron/MSC scaffolds demonstrated a significantly higher GAG production per DNA when compared to their corresponding chondron-only groups [7]. Additionally, we found type II collagen deposition in all groups, but the chondron/MSC scaffolds showed a more intense proteoglycan staining.

Efficacy of our one-stage cell therapy was confirmed using goats as preclinical animal models (*n* = 8 goats; *n* = 16 knees) [7]. Within the medial femoral condyles, a bilateral 5-mm full-thickness cartilage defect was created. One knee was treated with autologous chondrons and allogeneic MSCs, and the contralateral knee underwent the microfracture procedure, which was the clinical gold standard at the time (2010) [7]. Prior to this study, MSCs were harvested and isolated from bone marrow taken from the iliac crest of donor goats which were then cultured expanded and cryopreserved [7]. If a knee received a microfracture procedure, the subchondral bone was exposed and then perforated using a K-wire to allow marrow to fill the defect. If a knee received chondrons combined with MSCs cell therapy, the rapid digestion protocol was used to isolate chondrons from the debrided cartilage tissue within the defect and combined with the goat MSCs in a 10% chondron/90% MSCs mixture within a fibrin scaffold. After 6 months, the microfracture treated defects were filled incompletely, while the knees receiving IMPACT were almost completed filled with cartilage-like tissue [7]. In addition, histological scores were higher in the IMPACT group, as well as GAG content corrected for DNA [7]. 

Notably, there was no ectopic or uncontrolled growth which was observed in the nude mice. We also didn’t observe any safety concerns in the goats which was the first implantation of allogeneic cells in non-immunocompromised animals. Additionally, we didn’t see signs of inflammation, rejection, or reaction to the cell project. These in vivo data indicated that the combined IMPACT cell products were superior in repairing cartilage defects and promoting hyaline-like cartilage tissue than microfracture.

### Clinical Trials

The consilience of in vitro and in vivo data led us to conduct a first in-human clinical trial using the IMPACT cell therapy in single-stage surgical procedure for acute cartilage defects. This phase investigator-initiated I/II clinical trial was conducted at the University Medical Center Utrecht and treated 35 patients who sustained a full thickness cartilage defect using allogeneic bone-marrow derived MSCs and recycled autologous chondrons (clinicaltrials.gov ID: NCT02037204) [9, 10]. At a mean follow-up of 5 years, we demonstrated that IMPACT was safe and resulted in significantly improved patient’s symptoms and overall function, and a reduction in pain which was durable [20]. Additionally, one year after the procedure, we did a second look arthroscopy and biopsy of the healed cartilage in these patients. The macroscopic and histological evaluation of the tissue found hyaline articular cartilage [10]. The magnetic resonance imaging results demonstrated mean t1rho values that were not significantly different between the repaired tissue and the adjacent healthy cartilage at this one-year time point [10]. Moreover, upon DNA analysis of the tissue, we found that the DNA from the allogenic MSCs was no longer present in the healed cartilage and the repaired tissue only contained the patient’s DNA [10]. This demonstrates the safety and efficacy of using allogeneic MSCs to stimulate the repair of autologous chondrons and that a single-stage cell therapy is possible for articular cartilage repair. Currently, we are continuing our studies on IMPACT with the IMPACT2 clinical trial (clinicaltrials.gov ID: NCT04236739) in which we are randomizing patients to either our IMPACT cell therapy or a 9-month conservative treatment consisting of physical therapy and pain medication [11]. After the 9-months, patients who were randomized to the conservative treatment can also receive the IMPACT cell therapy [11]. The early unpublished IMPACT2 data demonstrate that IMPACT treatment shows superior outcomes compared to non-surgical treatment.

After receiving Federal Drug Administration Investigational New Drug approval, our research team recently completed another clinical trial at Mayo Clinic. In this, allogeneic adipose-derived MSCs and autologous chondrons were used to treat focal chondral lesions. As mentioned previously, the use of adipose-derived MSCs was strategic because these cells were easier to harvest from patients undergoing liposuction procedure and there was a higher yield of MSCs from adipose tissue than bone marrow. In addition, our unpublished clinical outcomes at 2 years are similar to the clinical outcomes in the first European clinical trial which utilized bone-marrow derived MSCs. Together, these data strongly suggest that combined cell therapy improves clinical outcomes and that the source of MSCs may not be important if we are using them as signaling cells. This makes our combined two cell-type therapy the first patient specific one-step, cell-based cartilage repair procedure that has been validated in patient clinical trials in both Europe and the United States.

### A Cell Therapy Platform

This review summarized our research group’s efforts to develop a one-stage, two cell-type surgical procedure for articular cartilage repair moving from the bench to bedside. Our group has also conducted studies to understand the synergistic mechanism of combining autologous chondrons and allogeneic MSCs. During in vitro chondron and MSC co-culture experiments, we have discovered that there is an exchange of mitochondria between the cells [21]. This mitochondrial transfer occurs within the first few hours and occurs via tunneling nanotubes, direct cell-to-cell contact, and extracellular vesicles [21]. To further explore this concept, we isolated mitochondria from MSCs, transferred them to chondrocytes, and conducted pellet cultures for approximately 28 days [21]. In our analysis, we found that DNA content and proteoglycan deposition were greater in the pellet cultures that had received MSC mitochondria when compared to the control groups without added MSC mitochondria [21]. Furthermore, there was an increase in MSC mitochondrial DNA over time. Interestingly, when we analyzed the biopsies from our phase I/II human clinical trial, no donor mitochondrial DNA was traceable [21]. Mitochondrial transfer might not occur in vivo, or donor mitochondria are cleared from the cartilage within the first year after transplantation [21]. These data indicate that mitochondrial transport may be one of the mechanisms by which our IMPACT/RECLAIM procedure results in authentic articular cartilage repair. However, further studies are needed to understand the role of mitochondrial transfer in musculoskeletal tissue repair.

Moreover, we have shown that MSCs may have anti-inflammatory and chondroinductive roles when combined with other cells. In a recent study, we evaluated whether extracellular vesicles secreted by the bone marrow MSCs used in the clinical study impact human osteoarthritic chondrocytes [22]. We found that the MSC-derived extracellular vesicles inhibit the adverse effects of inflammatory mediators on osteoarthritic chondrocytes [22]. When we performed co-cultures with isolated inflammatory-licensed OA chondrocytes, the MSC extracellular vesicles inhibited the inflammatory and catabolic response [22]. In addition, these extracellular vesicles promoted cartilage regeneration by stimulating the production of proteoglycans and type II collagen, and decreasing the expression of hypertrophic marker genes [22]. We also conducted a study to understand the impact of direct cell-cell contact between MSCs and chondrocytes [14]. In this study, we found that MSCs stimulate cartilage formation when in contact or close proximity to chondrocytes and that direct cell-cell contact and communication through gap junctions are important for chondrogenesis [14]. Overall, the results indicate that MSCs stimulate cartilage production when in close proximity to chondrocytes and that direct cell to cell contact and communication through gap junctions has an additional effect to the MSC chondroinductive mechanism [14]. 

Importantly, the underpinnings of our cell therapy which uses allogeneic MSCs as signaling cells to promote endogenous tissue repair, provides a technique that could be applied to other joints and musculoskeletal applications [23]. We have further expanded our work to chondral defects within the hip joint for which we are currently conducting a phase 1 clinical trial (clinical trials.gov ID: NCT05553132). Please see a summary of clinical trials using our cell therapy platform and its future directions in Table 1. Furthermore, our group is currently pursuing studies applying this technique to acute meniscus tears and torn rotator cuff tendons. While these tissues present other unique challenges, we anticipate the allogeneic MSCs to have a similar impact on meniscus cells and tenocytes. In a series of manuscripts, we have demonstrated that MSCs can be used within meniscus tissue to promote tissue repair and revitalize meniscus allografts [24–27]. In addition, MSCs can stimulate isolated meniscus cells to produce meniscus tissues at similar ratio’s as found for chondrocytes and chondrons [26]. The surgical feasibility of using a combination of meniscus cells and MSCs in a meniscus scaffold was shown in a cadaver study [27]. Our studies are just beginning for rotator cuff repair, but we have demonstrated that there are multiples sources of tissues to recycle cells from including rotator cuff tendons, biceps tendon, and bursa tissue. We anticipate this combined cell therapy will be a platform cell therapy with a wide range of musculoskeletal applications.

**Table 1:** A summary of clinical trials using the IMPACT/RECLAIM cell therapy platform and future directions

Clinical Trial	Trial registration	Brief Description of Trial Design		Participants	Study Location	Publication Reference
IMPACT (2013–2014)	NCT02037204	First in man feasibility and safety study Europe	Single arm study allogeneic bone marrow derived MSCs with autologous recycled chondrons for knee cartilage defects (Phase I/II)	35	Netherlands	De Windt 2017 and 2017, Saris 2021^1–3^
IMPACT2 (2019–2022)	NCT04236739	Efficacy of IMPACT compared to nonsurgical care	Phase III crossover RCT of allogeneic bone marrow derived MSCs with autologous recycled chondrons vs. conservative care for knee cartilage defects (Phase III)	60 (planned) 50 (current, ongoing)	Netherlands	Korpershoek 2020^4^– collecting data in follow up
Knee RECLAIM (2018–2022)	NCT03672825	First in man feasibility and safety study USA	Single arm study allogeneic adipose tissue derived MSCs with autologous recycled chondrons for knee cartilage defects (Phase I/II)	25	United States	Saris 2022^5^
Hip RECLAIM (2022-)	NCT05553132	Safety and feasibility study for Hip Cartilage Defects	Single arm study allogeneic adipose tissue derived MSCs with autologous recycled chondrons for hip cartilage defects (Phase I/II)	15	United States	Collecting data in follow up
Shoulder RECLAIM						

### Key References

- Wu L, Leijten J, van Blitterswijk CA, Karperien M. Fibroblast growth factor-1 is a mesenchymal stromal cell-secreted factor stimulating proliferation of osteoarthritic chondrocytes in co-culture. Stem Cells Dev. 2013;22(17):2356-67. This study is some of the first evidence to demonstrate that co-culturing MSCs with chondroctyes stimulates chondrocyte proliferation.
- Bekkers JE, Tsuchida AI, van Rijen MH, Vonk LA, Dhert WJ, Creemers LB, et al. Single-stage cell-based cartilage regeneration using a combination of chondrons and mesenchymal stromal cells: comparison with microfracture. Am J Sports Med. 2013;41(9):2158-66. This manuscript established that a single-stage cartilage repair procedure that combines allogeneic MSCs and autologous chondrons demonstrates better cartilage repair tissue using both in vitro and in vivo studies.
- de Windt TS, Vonk LA, Slaper-Cortenbach IC, van den Broek MP, Nizak R, van Rijen MH, et al. Allogeneic Mesenchymal Stem Cells Stimulate Cartilage Regeneration and Are Safe for Single-Stage Cartilage Repair in Humans upon Mixture with Recycled Autologous Chondrons. Stem Cells. 2017;35(1):256 − 64. This paper highlighted that allogenic MSCs are safe to use in patients, improves patient clinical outcomes compared to baseline, and second look arthroscopy and biopsy demonstrated hyaline-like cartilage regeneration.
- de Windt TS, Vonk LA, Slaper-Cortenbach ICM, Nizak R, van Rijen MHP, Saris DBF. Allogeneic MSCs and Recycled Autologous Chondrons Mixed in a One-Stage Cartilage Cell Transplantion: A First-in-Man Trial in 35 Patients. Stem Cells. 2017;35(8):1984-93. This study evaluated the tissue taken from the second look biopsy and found through DNA short tandem repeat analysis that the regenerated tissue contained patient-DNA only.
- Saris TFF, de Windt TS, Kester EC, Vonk LA, Custers RJH, Saris DBF. Five-Year Outcome of 1-Stage Cell-Based Cartilage Repair Using Recycled Autologous Chondrons and Allogenic Mesenchymal Stromal Cells: A First-in-Human Clinical Trial. Am J Sports Med. 2021;49(4):941-7. This manuscript showed mid-term safety and efficacy of the cell therapy which combined allogeneic MSCs and autologous chondrons. The patients demonstrated durable improvement in clinical outcomes from baseline and no serious adverse events were noted.

## Data Availability

No datasets were generated or analysed during the current study.
